# Death of Dr. Coleman Rogers

**Published:** 1855-02

**Authors:** 


### DEATH OF DR. COLEMAN ROGERS.

Dr. Coleman Rogers, the oldest and one of the most eminent of the physicians of Louisville, died at his residence on the 17th inst. Dr. Yandell, in announcing the event to the medical class of the University of Louisville, made the following remarks on his life and character, which form as full a biographical sketch of the deceased as our time and limits will permit at present:

Gedtlemen: Another patriarch of our profession, a cotemporary of Chapman, Drake, Caldwell, and Cooke, is gone. Dr. Coleman Rogers, the father of your beloved teacher, who, but for this melancholy event, would have been on this stand before you at this hour, the most aged and one of the most eminent of our physicians, departed this life a few hours ago. He had been long a practitioner of Louisville, and was known to almost every citizen as one who in the prime of life had stood in the foremost rank of his profession.

Dr. Rogers was a native o' Virginia, but came with his father, at a very early age, to Kentucky, and was reared in the neighborhood of Lexington. He studied medicine with that accomplished physician and gentleman, Dr. Samuel Brown, and subsequently attended medical lectures in the University of Pennsylvania, at a time when the fame of Rush was at its zenith, when Wistar was in his prime, and Physick was without a rival in surgery in all our broad land. After attending two courses of lectures he was compelled to return home before taking his degree, in the spring of 1805, but graduated in that venerable institution several years later. During his first residence in Philadelphia he was a private pupil of Dr. Charles Caldwell, who was then industriously laying the foundation of that renown which afterwards became co-extensive with our country. Dr. ROGERS first settled in Danville, where he was associated in practice with the distinguished surgeon, Dr. EPHRAIM MCDOWELL. Removing after a few years to the neighborhood of Lexington, in one of the early attempts to organize the medical department of Transylvania University, he was elected Adjunct Professor of Anatomy and Surgery. This honor, however, he declined, probably deeming the enterprise of founding at that time a medical school in the western wilds of America premature. It was several years later before the medical department of that earliest of our literary institutions was put into successful operation. Dr. ROGERS, in the mean time, had removed to Cincinnati, where he pursued his profession for a number of years, a portion of the time in partnership with Dr. DRAKE, who like himself, had been brought up in Kentucky. He participated with Dr. DRAKE in the infant enterprise of medical teaching in Cincinnati, and for a single season was connected with the Medical College of Ohio as professor of Surgery. But he was not satisfied with a residence out of the State where he had spent his early years. In 1823 he removed to Louisville, where he has since continued to reside until the summons came, this morning, calling him to his final rest.

When the Medical Institute of Louisville was chartered, in 1833, Dr. ROGERS was elected by the Managers professor of surgery, but as the organization was not completed at that time he performed no public duties under the appointment. Four years later the Institute was reorganized, and during the progress of the movement he showed himself one of the warmest and most disinterested friends of the enterprise. The chair of surgery was again tendered to him, but he finally declined the honor, concluding, no doubt, that he had reached a period in life too advanced to enter successfully upon a new career. But he was nevertheless as zeal ous a promoter of the undertaking as if he had embarked everything in its fortunes, and watched the progress of the school during its infancy with almost a paternal solicitude.

Dr. ROGERS, ever since I had the honor of knowing him, has been a student, and there can be no doubt that he formed those habits of study early in life. He kept pace with the progress of his profession in its accelerated march. No addition was made to the store of its practical facts that he did not soon possess himself of it; no change occurred in its philosophy with which he was not soon acquainted. He was a constant, daily, interested reader on medical subjects. If a new book appeared, you were sure to find that Dr. Rogers had read it and mastered its contents, for he was not a mere superficial reader to take up indiscriminately whatever was thrown out, but his mind was of that independent cast that weighed and analysed what he read. These habits of application to study, and this undivided devotion to his profession through a long life are worthy of all praise, and should awaken a spirit of emulation in those who are pursuing the same vocation. They rendered Dr. Rogers a thorough physician early in life, and kept him prominent among the foremost in his profession to its close. His faculties took no rust for they were kept in active exercise. With declining powers of body, with the sense of sight impaired and the sense of hearing almost gone, he continued to toil on in the discharge of professional duties to the last, and only relinquished his habits of study with his life.

Dr. Rogers was a man of strong mind and an iron will, warm and steady in his attachments, and inflexible in his purposes. The profession of our city mourns him to-day as a pioneer and patriarch, who, for nearly half a century, labored faithfully to maintain the interests and dignity of his calling. Dr. Rogers was in the 74th year of his age.

